# A Novel Ensemble Artificial Intelligence Approach for Gully Erosion Mapping in a Semi-Arid Watershed (Iran)

**DOI:** 10.3390/s19112444

**Published:** 2019-05-29

**Authors:** Dieu Tien Bui, Ataollah Shirzadi, Himan Shahabi, Kamran Chapi, Ebrahim Omidavr, Binh Thai Pham, Dawood Talebpour Asl, Hossein Khaledian, Biswajeet Pradhan, Mahdi Panahi, Baharin Bin Ahmad, Hosein Rahmani, Gyula Gróf, Saro Lee

**Affiliations:** 1 Geographic Information Science Research Group, Ton Duc Thang University, Ho Chi Minh City, Vietnam; buitiendieu@tdtu.edu.vn; 2 Faculty of Environment and Labour Safety, Ton Duc Thang University, Ho Chi Minh City, Vietnam; 3 Department of Rangeland and Watershed Management, Faculty of Natural Resources, University of Kurdistan, Sanandaj 66177-15175, Iran; k.chapi@uok.ac.ir; 4 Department of Geomorphology, Faculty of Natural Resources, University of Kurdistan, Sanandaj 66177-15175, Iran; d.talebpoor@gmail.com; 5 Department of Rangeland and Watershed Management, Faculty of Natural Resources and Earth Sciences, University of Kashan, Kashan 87317-53153, Iran; ebrahimomidvar@kashanu.ac.ir; 6 Institute of Research and Development, Duy Tan University, Da Nang 550000, Vietnam; phambinhgtvt@gmail.com; 7 Kurdistan Agriculture and Natural Resources Research and Education Center, AREEO, Sanandaj 66169-36311, Iran; hkhaledian@yahoo.com; 8 Center for Advanced Modeling and Geospatial System (CAMGIS), Faculty of Engineering and IT, University of Technology Sydney, CB11.06.106, Building 11, 81 Broadway, Ultimo NSW 2007, Australia; Biswajeet.Pradhan@uts.edu.au; 9 Department of Energy and Mineral Resources Engineering, Choongmu-gwan, Sejong University, 209 Neungdong-ro, Gwangjin-gu, Seoul 05006, Korea; 10 Department of Geophysics, Young Researchers and Elites Club, North Tehran Branch, Islamic Azad University, Tehran P.O. Box 19585/466, Iran; panahi2012@yahoo.com; 11 Faculty of Built Environment and Surveying, Universiti Teknologi Malaysia (UTM), Johor Bahru 81310, Malaysia; baharinahmad@utm.my; 12 Department of Computer Science and Engineering, and IT, School of Electrical and Computer Engineering, Shiraz University, Shiraz 84334-71964, Iran; hosein.rhm@gmail.com; 13 Department of Energy Engineering, Budapest University of Technology and Economics, Budapest 1111, Hungary; grof@energia.bme.hu; 14 Geoscience Platform Research Division, Korea Institute of Geoscience and Mineral Resources (KIGAM), 124 Gwahak-ro Yuseong-gu, Daejeon 34132, Korea; 15 Department of Geophysical Exploration, Korea University of Science and Technology, 217 Gajeong-ro Yuseong-gu, Daejeon 34113, Korea

**Keywords:** gully erosion, machine learning, ensemble algorithms, geomorphology, Geographic information science, Kurdistan province

## Abstract

In this study, we introduced a novel hybrid artificial intelligence approach of rotation forest (RF) as a Meta/ensemble classifier based on alternating decision tree (ADTree) as a base classifier called RF-ADTree in order to spatially predict gully erosion at Klocheh watershed of Kurdistan province, Iran. A total of 915 gully erosion locations along with 22 gully conditioning factors were used to construct a database. Some soft computing benchmark models (SCBM) including the ADTree, the Support Vector Machine by two kernel functions such as Polynomial and Radial Base Function (SVM-Polynomial and SVM-RBF), the Logistic Regression (LR), and the Naïve Bayes Multinomial Updatable (NBMU) models were used for comparison of the designed model. Results indicated that 19 conditioning factors were effective among which distance to river, geomorphology, land use, hydrological group, lithology and slope angle were the most remarkable factors for gully modeling process. Additionally, results of modeling concluded the RF-ADTree ensemble model could significantly improve (area under the curve (AUC) = 0.906) the prediction accuracy of the ADTree model (AUC = 0.882). The new proposed model had also the highest performance (AUC = 0.913) in comparison to the SVM-Polynomial model (AUC = 0.879), the SVM-RBF model (AUC = 0.867), the LR model (AUC = 0.75), the ADTree model (AUC = 0.861) and the NBMU model (AUC = 0.811).

## 1. Introduction

Water-related soil erosion as an environmental concern and considerable source of transferring sediments into rivers is a threating land degradation phenomenon affecting around one billion hectares in the world [1]. The consequents of the water erosion include on-site impacts such as loss of soil resources, decrease in soil fertility, reduction of vegetation growth, filling of valleys and reservoirs, desertification and destruction of human infrastructure, and off-site impacts consisting of sedimentation of water courses, decreases in water quality and economic and ecological damages to societies [2,3]. Water erosion occurs in different forms based on changes in its morphometric characteristics on hill slopes including rain splash, sheet (interrill) erosion, rill erosion, bank erosion and gully (badland) erosion [4]. Among these, gully erosion is a complex erosion problem [5] that will be accelerated or triggered with land use change and heavy rainfalls [6]. The contribution of gullies in overall sediment production in semi-arid and arid regions is 50–80% worldwide [7]. It has been reported that soil loss rates by gully erosion ranges from minimal 10% up to 94% of total sediment yield in water erosion [5].

According to the definition, gully erosion is an erosion process where runoff water accumulates and sometimes recurs in narrow channels and then, over a short time, the soil from the narrow channels will be removed and a considerable channel with high depth will emerge [8]. Three types of gullies have been reported as (i) permanent gullies which are often related to agricultural lands, and are specified with very deep channels that with ordinary tillage are obliterated. Their depth ranges from 0.5 to as much as 25–30 m [9,10,11,12], (ii) ephemeral gullies (rill form) are small eroded channels by overland flow that are easily filled through normal tillage [9]. They are specified by a critical cross-sectional area of about 929 cm^2^ [13], a minimum width and depth of 0.3 and 0.6 m, respectively [14], and (iii) bank gullies constitute wherever a morphological bank will be cut by concentrated runoff. With increasing the local slope of the soil surface as subvertical or vertical, they will be quickly developed by erosion, piping and consequently mass movements at or below the soil surface [15,16].

An area of about 1.1% of the world’s land areas has been covered by Iran where the annual amount of soil loss is 2–2.5 billion tons, ranking as the second in the world in terms of the amount of soil erosion [17]. Reports indicate that about 88 million hectares (more than half of the area) of Iran is covered by critical soil erosion conditions [17]. Since gullies lead to degradation of a large amount of soil and transferring huge volume of sediments into streams, the agriculture lands, residential areas and even infrastructures will suffer [18]. Therefore, recognition of the areas that are more prone to gully erosion is a critical issue for better land management and prevention of gully erosion in land allocation studies.

Over the last decades, some investigations and numerous computer-aided techniques have been developed for gully erosion modelling including expert knowledge methods such as analytical hierarchy process (AHP) [19,20], bivariate statistical methods (BSMs) such as frequency ratio (FR) [21,22], certainty factors (CF) [23], weight of evidence (WoE) [22,24], information value (InVal) and evidential belief function (EBF) [25], conditional probability (CP) [26], index of entropy (IOE) [27], multivariate statistical methods (MSMs) such as linear regression (LiR) [28] and logistic regression (LR) [29,30], and machine learning methods such as support vector machine (SVM) [31,32,33], and random forest (RF) [34,35], classification and regression trees (CART) [33] and artificial neural networks (ANN) [33].

Recently, ensemble machine learning methods have been used more for spatial prediction of natural hazards studies such as groundwater and flood [36,37,38,39,40,41,42,43,44], landslides [45,46,47,48,49,50,51,52,53,54,55,56,57,58], wildfire [59], sinkhole [60], droughtiness [61] and land/ground subsidence [62]. However, few studies using ensemble machine learning models have been reported on gully erosion, such as [31]. An advantage of the ensemble algorithms as powerful techniques is that they have higher goodness-of-fit and perdition accuracy than the individual or single-based methods/algorithms by removing their weaknesses. For instance, Pourghasemi et al. [31] used artificial neural network (ANN), SVM, maximum entropy (ME) and their ensembles to prepare gully susceptibility mapping. They reported that the ANN-SVM ensemble had more ability to detect gully erosion in comparison to the individual and other ensemble methods. Although some methods and techniques have been developed for susceptibility assessment, the results of the modeling process are different from one region to another even from a model to another one, indicating that the obtained results by a model are for that specific case study. Overall, ensemble machine learning has improved the prediction capability of gully erosion models significantly.

This aim of this work is to expand the body of the proposed gully erosion modelling and verify a new ensemble artificial intelligence approach based on rotation forest (RF) and the ADTree algorithm, named as RFADT, for gully erosion mapping in a semi-arid watershed, Klocheh, Bijar in Kurdistan province, Iran. RF is a relatively new and powerful ensemble framework that has proven its efficiency in various real world problems [63,64,65,66,67], whereas the ADTree is a new robust and efficient algorithm [41]. To the best of our knowledge, RFADT has not been considered for gully erosion susceptibility mapping. Finally, the effectiveness of the proposed RFADT model is assessed by comparing its performance with benchmarks, ADTree, SVM with RBF and Polynomial kernel functions, LR and Naïve Bayes Multinomial Updatable (NBMU), and conclusions are given.

## 2. Description of Study Area

**Figure 1:**
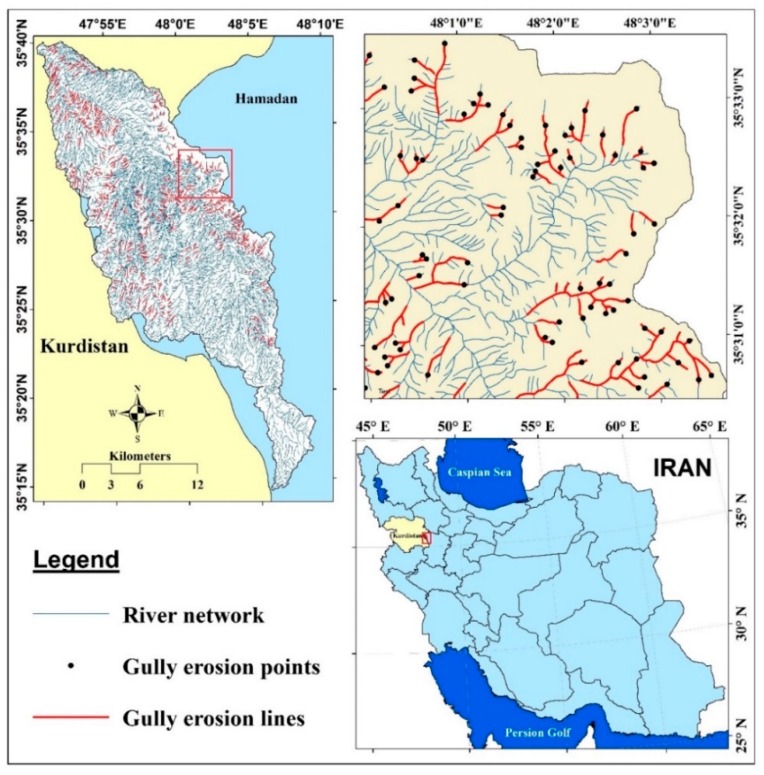
Location of study area and gully erosion sites in Kurdistan province and Iran.

The study area is the Klocheh watershed, located between Kurdistan province and Hamadan province in the west of Iran, between longitudes 47°50′24″ E and 48°8′35″ E, and latitudes 35°14′24″ N and 35°40′5″ N, covering an area of about 498.49 km^2^ (Figure 1). Elevation in the study area varies from 1612 to 2331 m above sea level, with an average elevation of 1925.47 m. The terrain surface of the area is relatively steep with slope ranging from 0° to 67.06° with the mean slope of 6.56°. Statistical analysis of rainfall for the period of 1987–2010 shows that the annual average rainfall is about 338 mm. The mean daily maximum and minimum temperatures are 4.4 and 13.4 °C, respectively. The number of frost days is 104 and the number of snow days is 35 (http://kurdistanmet.telepol.ir).

The Klocheh watershed is a branch of the Sefid Rood River which the latter itself is drained into the Caspian Sea Basin. Six geomorphologic units can be identified in the Klocheh watershed including old plain unit (40.43%), new plain unit (29.98%), hill slope unit (21.38%), fluvial sediment unit (3.72%), valley unit (3.20%) and mountain unit (1.28%). In this study, five types of land use patterns were also identified including barren lands, dry farming lands, poor pasture lands, semi-dense pasture lands and woodlands. The dry farming lands have occupied the largest area (73.95%), followed by semi-dense pasture (11.97%), poor pasture (9.58%), woodlands (3.38) and barren lands, respectively. The Klocheh watershed is geologically located in the Sanandaj-Sirjan zone so that its effects are seen as magmatism in the basin (geological map with scale of 1:100,000). The lithology of the basin includes metamorphic-sedimentary rocks of the Jurassic period and Tertiary sediments includes Js (schist, sandstone, quartzite), JL (intracellular limestone layers), Pl^t^ (trachyte, trachyandesite, dacite) and Pl^b^ (basalt, basinite), which are covered by deposits of Mm (light green and red marls), P^cg^ (conglomerate loose deposits), P^l,m^ (clay limestone, marl, sand marl, limestone sandstone), Qt^1^ (high alluvial sediments) and Qa^l^ (river beds sediments) (geological map with scale of 1:100,000). Stone units cover about 4.93% and sediment units cover 95.07% of the basin surface. Based on this classification, sedimentary and rocky units of the basin have been classified into low erodibility units such as Js, Pl^t^, and JL; moderate erodibility including Q^c^, Qt^1^, P^cg^, Mm, and Pl^b^; high erodibility including Qt^r^, and Qt^2^; very high erodibility including Qa^l^, Pl^m^, covering an area of about 4.061%, 43.826%, 20.49% and 31.62% of the basin, respectively. Gullies in the study area have been mainly formed due to susceptible lithological units such as marl and alluvial deposits on the rivers. We in this study selected the head of gullies of the tributaries of the streams. The gullies on the main river of the study area had large sizes in depth (>10 m) and width (>7 m) while the head of gullies of the tributaries had smaller sizes (depth < 2–3 m and width < 1 m). Our main aim of this study was to recognize the locations that are prone to gully development in the future.

## 3. Data Acquisition

### 3.1. Gully Inventory Map

**Figure 2:**
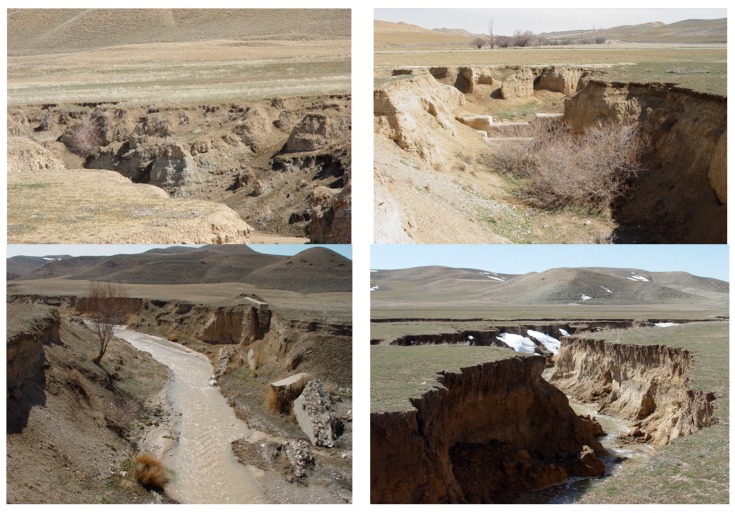
Photos of gully erosion in the Klocheh Watershed, Kurdistan province, Iran.

In this study, locations of some gullies had been recorded earlier by the Natural Resources and Watershed Management General Office of Kurdistan province; however, other locations were recorded during comprehensive field surveys and these locations were then checked by Google Earth images (dated 22 May 2017) in order to prepare an accurate gully erosion inventory map. A total of 915 gully erosion lines were ultimately detected in the study area which were mainly on or near the river networks (Figure 1). These gully lines were converted to the points using “feature to point” using ArcGIS 10.2, with more focus on the head of gullies. These points were then randomly classified into 70% (640 gullies) and 30% (275 gullies) for modeling and validation processes, respectively. Most of the gullies are classified as permanent and bank gullies (stream gullies) in the current study. It should be noted that for the modeling process using machine learning algorithms, the dataset should contain both present and absent events of the gully erosion process. Basically, besides dividing gully erosion locations into 70% and 30%, a total of 915 non-gully erosion locations should be selected and classified into a ratio of 70%/30%. In this study, we selected these locations randomly over the watershed using “create random point” tool in ArcGIS 10.2. Figure 2 shows some typical examples of gullies in the study area. As can be seen in these figures, gullies in the study area are surprisingly developed from rill with small size in depth and width, in which some of them have a depth more than 4 m and a width more than 10 m. The primary filed surveys based on the expert knowledge revealed that shear stress of flow and geology were the most important factors to cause gullies. Indeed, the gully locations are in concordance with the loose and erodible quaternary depositions including marl with interlayers of limestone. The Natural Resources and Watershed Management General Office of Kurdistan province, Iran, has done many control practices including construction of check dams to prevent and even to control these gullies; however, as observed in Figure 2, all of them were unsuccessful since the check dams were destroyed and overturned.

### 3.2. Gully Erosion Conditioning Factors

A large set of geo-environmental factors are usually used in scientific literature to analyze gully erosion hazard. However, there are no universal guidelines for selecting gully conditioning factors. Previous researchers have considered different factors as independent variables. According to the literature, we selected 22 gully-erosion susceptibility predictor variables, which can be divided into six categories (Table 1): (1) Topographic factors; (2) Hydrological factor; (3) Lithological factors; (4) Land cover factors; (5) Anthropogenic factors; and (6) Geomorphological factors. Topographic factors include slope, aspect, elevation, plan curvature, profile curvature, sediment transport index (STI) and valley depth (VD). Hydrological factors include rainfall, stream power index (SPI), topographic wetness index (TWI), hydrological group (HG), flow accumulation, permeability, distance to river and river density. Lithological factors refer to lithology, distance to fault and fault density. Land cover factors include land use while distance to road and road density factors are anthropogenic factors considered in the analysis; and geomorphological factors enclose landforms. Table 1 shows gully conditioning factors and their classes for gully erosion modeling.

A Digital Elevation Model with 12.5 m resolution was extracted from ALOS PALSAR data, collected from Alaska Satellite Facility’s (https://vertex.daac.asf.alaska.edu/#). Slope, aspect, elevation, plan curvature, STI, VD, SPI, TWI, HG, flow accumulation, permeability, distance to river and river density were constructed from the digital elevation model (DEM) using ARC GIS 10.2 and SAGA 6.0.0 software.

Slope inclination is an important factor in gully formation and development. Gentle slopes are assumed to have higher infiltration in comparison to steeper slopes, and therefore gentle slopes are considered to be susceptible to gully initiation [21,68]. The slope factor was classified into six classes of (1) 0–2; (2) 2–5; (3) 5–10; (4) 10–15; (5) 15–20; (6) >20 (Table 1). Aspect is another conditioning factor that plays an important role in gully development [26]. Aspect can control evapotranspiration, vegetation cover and incoming solar radiation [69]. The aspect factor of the study area was created using the DEM and categorized later into nine classes of (1) Flat; (2) North; (3) Northeast; (4) East; (5) Southeast; (6) South; (7) Southwest; (8) West; (9) Northwest (Table 1). Elevation influences microclimate and vegetation community [70]. Therefore, several researchers have taken it into account in geohazards, especially for predicting gully-erosion susceptibility [30,71]. According to the previous research, most of occurred gullies were concentrated in low-altitude areas [26]. The elevation factor was divided to eight classes: (1) 1612–1700; (2) 1700–1800; (3) 1800–1900; (4) 1900–2000; (5) 2000–2100; (6) 2100–2200; (7) 2200–2300; (8) 2300–2400 m (Table 1). Plan curvature can be an important predictor of gully erosion by representing the spatial variability in diverging and converging overland flow of water [21,72]. The plan curvature factor of the study area was reclassified into five categories: (1) [(−5.67)–(−0.736)]; (2) [(−0.736)–(−0.188)]; (3) [(−0.188)–0.149]; (4) [0.149–0.697]; (5) [0.6974–5.08] (m^−1^) (Table 1). Profile curvature can reflect the geometric features of slopes, which in turn can influence stress distribution of slopes in the development of gully [73]. The profile curvature of the study area was classified into five classes: (1) [(−6.357)–(−0.972)]; (2) [(−0.972)–(−0.187)]; (3) [(−0.187)–0.317]; (4) [0.317–1.1]; and (5) [1.1–7.94] (m^−1^) (Table 1). The sediment transport index (STI) as another effective factor in gully erosion has an important role in characterizing the process of erosion and deposition. In the present study, the STI was divided into five classes: (1) 0–1.286; (2) 1.286–2.894; (3) 2.894–5.145; (4) 5.145–8.468; (5) 8.468–27.33 (Table 1). Valley depth (VD) is computed based on the elevation using SAGA 6.0.0 software. It was divided into five categories including (1) 0–48.231; (2) 48.231–108.520; (3) 108.520–176.340; (4) 176.340–254.720; and (5) 254.720–384.340 (Table 1).

Rainfall as a triggering factor by penetrating into the cracks of soils leads to gully occurrence and its development in different directions [6]. The annual average rainfall factor of the Klocheh watershed was obtained from the inside and outside rain gauge stations of the study area using Inverse Distance Weighted (IDW) method. The rainfall factor was divided into five classes including (1) 261–286; (2) 286–298; (3) 298–306; (4) 306–312; and (5) 312–322 mm.

Stream Power Index (SPI), as a hydrological factor, indicates the erosion power of streams that can affect gully occurrence [21]. It is calculated as follows [74]:

(1) $$\mathrm{SPI}=A_{s}\tan\beta ,$$

where A_s_ (m^2^m^−1^) is the specific catchment area and *β* is the cumulative upslope area and slope gradient (in degrees). The SPI factor of current study was divided into five classes including (1) 0–112.4; (2) 112.4–224.8; (3) 224.8–401.5; (4) 401.5–722.7; (5) 722.7–4095.

Topographic wetness index (TWI) is considered an important factor in gully development. Therefore, some researchers have applied TWI as a secondary topographic factor for modeling gully occurrence [26,30]. The formula of TWI is shown in Equation (2):

(2) $$\mathrm{TWI}=\ln\left(\frac{A_{s}}{\tan\beta }\right),$$

where A_s_ and β are the cumulative upslope area and slope gradient (in degrees), respectively. In this study, the TWI value was produced in SAGA-GIS 6.0.0 software using a 12.5 m DEM and then reclassified into five groups: (1) 1–3; (2) 3–4; (3) 4–5; (4) 5–6; (5) 6–9.059.

Hydrological soil group (HSG) is another conditioning factor in gully erosion studies. It reflects the soil potential for runoff generation based on the amount of infiltration [75]. The HSG factor was classified in four groups including (1) A; (2) B; (3) C; (4) D.

The distance to road map was constructed from the road network built by Iran National Cartographic Center (INCC) in DGN format with 1:25,000 scale. Flow accumulation, distance to river (m) and river density (km/km^2^) are prominent hydrological factors that have an important role in gully erosion. The possible effect of river networks on gully erosion was analyzed by calculating the distance to the nearest perennial or major upstream ephemeral rivers in the region in every raster cell. The values of three factors were constructed from the DEM 12.5 m using ArcGIS 10.2 and SAGA 6.0.0 software. Their classes are shown in Table 1.

Permeability or degree of porosity in soil indicates the ability of water to percolate and disintegrate the structure of soils [76]. It is expected that soils with low permeability and high pore spaces are more prone to gully occurrence. In this study, the permeability map was prepared by the constant-head test (ASTM D 2434). It was then classified into three categories including low permeability, moderate permeability and high permeability.

The different lithology and weathering properties of geologic parent materials influence land surface processes and development of erosional landforms such as gullies [21,68,77]. The lithology factor was obtained from a geological map with the scale of 1:100,000. Lithology units of the study area include layered limestone layers (JL); schist, quartzite, and dark gray metamorphosed sandstones (JS); (3) an alternative of light green and red marls (M^m^); (4) basalt and bazanite (PL^b^); (5) conglomerate with a matrix of marl and sandstone (P^cg^); (6) clay limestone, marl, sand marl, sandstone (Pl^m^); (7) trachyte, trachy-andesite, dacite (Pl^t^); (8) fluvial sediment (Qa^l^); (9) terraces land (Q^c^); (10) travertine stone (Qt^r^); (11) high alluvial terraces (Qt^1^); and (12) low alluvial terraces (Qt^2^) (Table 1).

Distances to fault (m) (proximity to the fault) and fault density (km/km^2^) (cumulative length of faults per unit area) are important lithological factors in gully erosion. The rills which are closer to faults or have higher cumulative length of faults in the area have higher probability of becoming gullies [22]. The distance to fault and fault density factors are extracted from a geological map with the scale of 1:100,000. They are classified into five classes that are shown in Table 1.

Land use is also a key element in land degradation in general and in gully formation in particular [68]. The land use map of the present study was exploited using interpretation of Landsat 7 ETM+ satellite images from the land cover map acquired on 25 August 2017. The land use factor was divided into five categories: (1) Wood lands; (2) Dry-farming and cultivated lands; (3) Poor pastures; (4) Semi-dense pastures; and (5) Destroyed pastures.

Distance to road (m) and road density (km/km^2^) as anthropogenic/man-made factors show a remarkable influence on gully erosion [78]. These two man-made factors were generated from a topographic map with the scale of 1:150,000. Then, they were divided into five categories shown in Table 1.

Geomorphologic units have different roles in gully erosion occurrence. For example, gullies will generally be formed on low slope angle and loose sediments (quaternary depositions). They will be triggered by changing in overland flow, decreasing in runoff lag time and increasing in runoff volume [79]. In this study, the geomorphological map was categorized into five classes including (1) The valley plain unit (2) Hilly unit; (3) Mountain unit; (4) New plain unit; (5) Old plain unit; and (6) Fluvial sediment unit (Table 1).

## 4. Background of Machine Learning Methods

### 4.1. Support Vector Machine Classifier

Support Vector Machine (SVM) which introduced by Vapnik [80], is a well-known machine learning classifier applied to facilitate the solution of many real world problems including landslide prediction [81,82], flood prediction [83,84] and forest fire prediction [85,86]. It is based on the principle of structural risk minimization of statistical learning theory to reduce the error test and complexity of computation. Using the SVM, an optimal hyper-plane is constructed to separate two classes for classification whereas one class is assigned as “1” located above the hyper-plane and another is assigned “0” located below the hyper-plane. A number of support vectors are used to define the optimal hyper-plane which can be obtained by minimizing the objective function as below:

(3) $$\mathrm{Min}\sum_{i=1}^{n}\varphi_{i}-\frac{1}{2}\sum_{i=1}^{n}\sum_{j=1}^{n}\varphi_{i}\varphi_{j}y_{i}y_{j}(x_{i},x_{j}).$$

Subject to

(4) $$\mathrm{Min}\sum_{i=1}^{n}\varphi_{i}y_{j}=0\;\mathrm{and}\;0\leq \alpha_{i}\leq D,$$

where $x=x_{i},i=1,2,...,n$ is a vector of input variables, $y=y_{j},j=1,2,...,n$ is a vector of output variables and *φ_i_* is defined as Lagrange multipliers.

At last, the decision function used for the classification can be expressed as below:

(5) $$f(x)=\mathrm{sgn}\left(\sum_{i=1}^{n}y_{i}\varphi_{i}K(x_{i},x_{j})+a\right),$$

where *a* is defined as the bias defined as the distance of hyper plane from the origin, $K\left(x_{i},\;x_{j}\right)$ are the kernel functions namely polynomial (POL) and radial basis function (RBF) which can be expressed as below [87]:

(6) $$K_{POL}\left(x_{i},\;x_{j}\right)={\left(\left(x.y\right)+1\right)}^{d},$$

(7) $$K_{RBF}\left(x_{i},\;x_{j}\right)=e^{-\gamma {\left\|x-x_{i}\right\|}^{2}}.$$

### 4.2. Logistic Regression Classifier

Known as the most popular multivariate statistical analysis, logistic regression (LR) has been applied to many scientific fields such as medical science [88,89], computer science [90] and natural hazard assessment [91,92]. It can be used for prediction and assessment of gully erosion in regional scale [30]. Main principle of LR is that it uses logistic function to analyze the relationship between a set of the conditioning factors based on a set of dependent variables and one or more independent variables. Logistic function used in the LR can be expressed as the following equations:

(8) $$Q=\frac{1}{1+e^{-t}}.\;,$$

(9) $$t=\log\;\mathrm{it}\;\left(a\right)=\ln\left(\frac{a}{1-a}\right)=e_{0}+e_{1}x_{1}+\ldots +e_{n\;}x_{n}.\;,$$

where Q is defined as the probability of an gully erosion occurrence, x_i_ (i = 1, 2, 3, …, n) are defined as the conditioning factors, t is defined as the linear logistic factor which varies from −∞ to +∞, e_0_ is defined as the constant modeling coefficient, e_i_ (i = 1, 2, 3, …, n) are the modeling coefficients, and n is defined as the number of independent variables.

### 4.3. Naïve Bayes Multinomial Updatable Classifier

Known as one of the effective Bayesian classifiers, Naïve Bayes has been applied to many studies such as text classification [93,94], heart disease prediction [95], classification of agricultural land soils [96], facies identification [97] and natural hazard prediction [98]. The main principle of naïve bayes (NB) is based on the probabilities of the observations from past observations to find the state of query among other variables in the dataset. It is a simple and fast learning method for classification. Training NB can be implemented through several steps such as (i) collection of data, (ii) estimation of the probability and mean for each class, (iii) crtion of the variance and covariance matrix and building of the discriminant function for each class. Decision function of NB can be expressed as the following equation:

(10) $$\begin{array}{l} y_{NB}\;=\;\arg\max\;P(y_{i})\;\prod_{i=1}^{n}P(x_{i},y_{i})\\ \;\mathrm{yi}=\left[\mathrm{gully},\mathrm{non}-\mathrm{gully}\right] \end{array}$$

where $x\;\left(x_{1},\;x_{2}\;,\ldots x_{n}\right)$ is the vector of the influencing factors and $y\;\left(y_{1},\;y_{2}\right)$ is the vector of the output variables (gully, non-gully), $P(y_{i})$ is defined as the prior probability of $y_{i}$, $P(x_{i},y_{i})$ is defined as the conditional probability expressed as below:

(11) $$P(x_{i},y_{i})\;=\;\frac{1}{\sqrt{2\pi \beta }}e^{\frac{-{\left(x_{i}-\alpha \right)}^{2}}{2\beta^{2}}},$$

where α and β are defined as the mean and standard deviation, respectively.

### 4.4. Alternating Decision Tree Classifier

Alternating Decision Tree (ADT) which introduced by Freund and Mason [99], is known as one of the effective decision tree classifiers which is based on the boosting algorithm. Representation of this classifier is to construct a classification tree where each decision node is replaced by two nodes such as a prediction node and a splitter node [100]. Out of these nodes, a prediction node is related with a real value and a splitter node is related with a test [101]. In the ADT, the decision rules are easy to be interpreted; therefore, its decision-tree structures are simpler than other decision classifiers such as Classification and Regression Tree (CART) [102] and Random forest [103]. Let a base ruler mapping to the real number from the instances includes a precondition *t*_1_ and a base condition *t*_2_ and *u* and *v* are two real numbers where the prediction is *u* as $t_{1}\cap t_{2}$ or *v* as $t_{1}\cap \bar{t_{2}}$ ($\bar{t}$ is a negation of *t*). Value of *u* and *v* can be calculated by the following equations:

(12) $$u=\frac{1}{2}\ln\frac{W_{+}\left(c_{1}\cap c_{2}\right)}{W_{-}\left(c_{1}\cap c_{2}\right)},$$

(13) $$v=\frac{1}{2}\ln\frac{W_{+}\left(t_{1}\cap {}^{-}t_{2}\right)}{W_{-}\left(t_{1}\cap {}^{-}t_{2}\right)},$$

where W(pr) is the total weight of the training instances which satisfy the predicate pr. The best precondition *t*_1_ and base condition *t*_2_ are chosen by minimizing the $Z\left(t_{1},t_{2}\right)$ which is expressed as follows:

(14) $$Z\left(t_{1},t_{2}\right)=2\sqrt{W_{+}\left(t_{1}\cap t_{2}\right)W_{-}\left(t_{1}\cap t_{2}\right)}+\sqrt{W_{+}\left(t_{1}\cap {\bar{t}}_{{}_{2}}\right)W_{-}\left(t_{1}\cap {\bar{t}}_{{}_{2}}\right)}+W\left({\bar{t}}_{{}_{2}}\right).$$

### 4.5. Rotation Forest Ensemble Classifier

**Figure 3:**
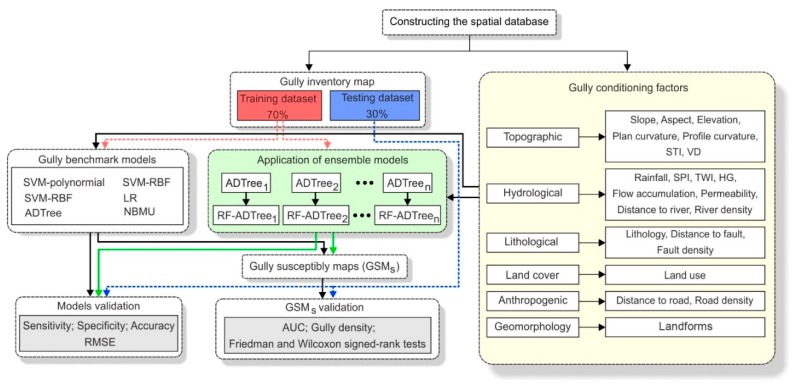
The flowchart of the study.

Proposed by Rodriguez [64], Rotation Forest (RF) is known as one of the most effective ensemble techniques which have been used for improving the predictive capability of many single classifiers such as naïve Bayes tree [45], Random forest [65], support vector machines [104]. Training the RF model can be carried out in several main steps such as (i) several subsets are generated by dividing the attribute sets, (ii) sample subsets are obtained by resampling and transforming features on the generated subsets, (iii) the rotation matrix is realigned according to sequence of original attribute sets, (iv) base classifiers are trained using the rotated sample subsets, and (v) the final outcome is obtained by integrating the results of various base classifiers on different sample subsets. In the RF, the rotation matrix is expressed as follows [64]:

(15) $$\begin{array}{l} R_{i}=\left[\begin{array}{cccc} e_{i,1}^{\left(1\right)},e_{i,1}^{\left(2\right)},\ldots ,e_{i,1}^{\left(M1\right)} & 0 & \cdots & 0\\ 0 & e_{i,1}^{\left(1\right)},e_{i,1}^{\left(2\right)},\ldots ,e_{i,1}^{\left(M2\right)} & \cdots & 0\\ \vdots & \vdots & \ddots & \vdots \\ 0 & 0 & \cdots & e_{i,1}^{\left(1\right)},e_{i,1}^{\left(2\right)},\ldots ,e_{i,1}^{\left(M_{K}\right)} \end{array}\right] \end{array},$$

where $e^{\left(1\right)}{}_{ij},e^{\left(2\right)}{}_{ij},\ldots ,e^{\left(Mj\right)}{}_{ij}$ are the coefficients of the rotation matrix, $M=\frac{n}{K}$ where *n* is the number of input factors and *K* is the number of subsets. Coefficients for each class in the given test sample are attained using the average combination method expressed as below [64]:

(16) $$\eta_{j}^{\left(X\right)}=\frac{1}{N}\sum_{i=1}^{N}c_{ij}(xR_{i}^{a}),\;j=1\ldots d.,$$

where $\eta_{j}^{\left(X\right)}$ is defined as the largest confidence of the output class, $c_{ij}\left(xR_{i}^{a}\right)$ is the probability assigned by the classifier with the regression $c_{ij}$, *d* is the number of output classes. The flowchart of this study is shown in Figure 3.

### 4.6. Factor Selection Using Information Gain Ratio (IGR)

Selecting the most important factors in the modeling process has a determinant role in the obtained results. In this stage, the factors that have noise and over-fitting problems will be detected and they should be eliminated from the final modeling process to achieve an accurate model [36,48]. There are some techniques for factor selection in the literature including Relief, Least Square Support Vector Machine (LSSVM), Fuzzy-Rough Sets (FRS), Information Gain, and Information Gain Ratio (IGR) [105]. Among these, the IGR technique [106] was used for selecting the most significant factors for gully erosion modeling using a training dataset. In this method, the IGR assigned the weights by entropy (En) method to each factor titled “average merit (AM)” and the factors will be ordered based on it. The higher the value of IGR is, the more important the conditioning factor will be. The AM is specified as the average information gain ratio with 10-fold cross-validation that has ranges between 0 and 1 [107]. Consider *T* as a training dataset with *n* input samples and the class label *G_i_* (gully erosion, non-gully erosion). The IGR will compute an AM for a given conditioning factor such as slope angle (SA) as follows [108]:

(17) $$IGR\;\left(T,\;SA\right)\;=\frac{E_{n}\left(T\right)-E_{n}\left(T,\;SA\right)}{SplitE_{n}(T,\;SA)},$$

(18) $$E_{n}(T)\;=\;-\sum_{i=1}^{2}\frac{n(A_{i},\;SA)}{|T|}\log_{2}\frac{n(A_{i},\;SA)}{|T|},$$

(19) $$E_{n}\;(T,\;SA)=\sum_{j=1}^{m}\frac{T_{j}}{|T|}E_{n}(T),$$

(20) $$SplitE_{n}(T,SA)=-\sum_{j=1}^{m}\frac{|T_{j}|}{|T|}\log_{2}\frac{|T_{j}|}{|T|}.$$

### 4.7. Development of Gully Erosion Maps

To construct the gully erosion maps each machine learning algorithm was performed based on each probability distribution function (PDF) of algorithms. Then, the gully erosion susceptibility indexes (GESI) for all pixels of the study area were computed. These values were converted to raster format using the “point to raster” tool in ArcGIS 10.2 and all gully erosion maps were prepared. Consequently, these maps were classified into five zones including very low susceptibility (VLS), low susceptibility (LS), moderate susceptibility, high susceptibility (HS) and very high susceptibility (VHS) using different classification methods such as equal interval, natural breaks, quantile and geometrical interval. In order to select the best classification method, the proportion of the whole cells of the watershed and all the observed gullies in each susceptibility class were calculated according to different classification methods and developed models.

### 4.8. Evaluation and Comparison Methods

#### 4.8.1. Statistical Index-Bases Measures

In this study, four statistical measures including sensitivity, specificity (SPF), accuracy and root mean square error (RMSE) were used for evaluation of the new proposed and other soft computing benchmark models. The sensitivity (SST), specificity (SPF) and accuracy (ACC) were computed based on the four types of possible consequences including True Positive (TP), False Positive (FP), True Negative (TN) and False Negative (FN) [109,110,111]. The TP and FP are the proportion of the number of gully cells that are correctly classified as gully and non-gully cells, respectively. While TN and FN are the number of gully cells classified correctly and incorrectly as non-gully cells, respectively. Basically, SST is defined as the number of correctly classified gully cells per total predicted gully cells. The SPF is the number of incorrectly classified gully cells per total predicted non-gully cells. While the ACC is the proportion of gully and non-gully cells which are correctly classified. The difference between the observed and estimated data can be obtained by the error metric of RMSE. The better performance of gully models were acquired when the values of SST, SPF, and ACC were high and the RMSE value was low. These statistical measures can be calculated as follows;

(21) $$SST=\frac{TP}{TP+FN},$$

(22) $$SPF=\frac{TN}{TN+FP},$$

(23) $$ACC=\frac{TP+TN}{TP+TN+FP+FN},$$

(24) $$RMSE=\sqrt{\frac{1}{n}}\sum_{i=1}^{n}(X_{P}-X_{A}{)}^{2},$$

where $X_{P}$ and $X_{A}$ are the predicted and actual (output) values in the training dataset or the validation dataset from the gully susceptibility models, and *n* is the total number of samples in the training dataset or the validation dataset.

#### 4.8.2. Receiver Operating Characteristic (ROC)

The Receiver Operating Characteristic (ROC) is a standard tool for evaluation the performance of the models that it is plotted based on the sensitivity and 100-specificity on the *x*- and *y*-axis, respectively [108]. The area under the ROC curve, AUC, generally has been used to evaluate model performance. The AUC for an ideal and inaccurate model have the values of 1 and 0.5, respectively [112]. The AUC is calculated as follows:

(25) AUC=(∑TP+∑TN)/(P+N),

where P and N are the total number of gullies and non-gullies, respectively.

#### 4.8.3. Freidman and Wilcoxon Sign Rank Tests

In addition to the abovementioned measures, two statistical tests including Freidman and Wilcoxon sign-ranked tests for more evaluation of the efficiency of the new proposed gully model were used. These non-parametric tests assess the comparison of performance of two or more gully models. If there are no differences between the treatment/performance of the gully models at the significant level of α = 0.05, the null hypothesis is predominant. To reject or accept the null hypothesis, the probability of a hypothesis (*p*-value) will be judged. The null hypothesis is rejected when it is true resulting in the existence of a significant difference between the two models and vice versa [108]. Freidman tests were used for evaluation of performance of models without pairwise comparison [113]. Consequently, if the *p*-value is less than 0.05 between two or more models (the null hypothesis is true), the results of comparison is not reliable [48]. Basically, Wilcoxon signed-ranked test is used to check the statistical significance of systematic pairwise between the gully models. The results by this test are judged based on the *p*-value and *z*-value if the *p*-value is less than 0.05 and the *z*-value exceeds the critical values of *z* (−1.96 and +1.96), the null hypothesis is true (rejected) and thus the performance of the susceptibility models is significantly different [45,108].

#### 4.8.4. Gully Density

Gully density for a gully erosion susceptibility map is defined as the ratio of the number of gully erosion cells to the number of cells in susceptibility class. It was computed for the machine learning algorithms and then the obtained results were analyzed and assessed.

## 5. Result and Analysis

### 5.1. The Most Important Factors in Gully Modelling by IGR

**Figure 4:**
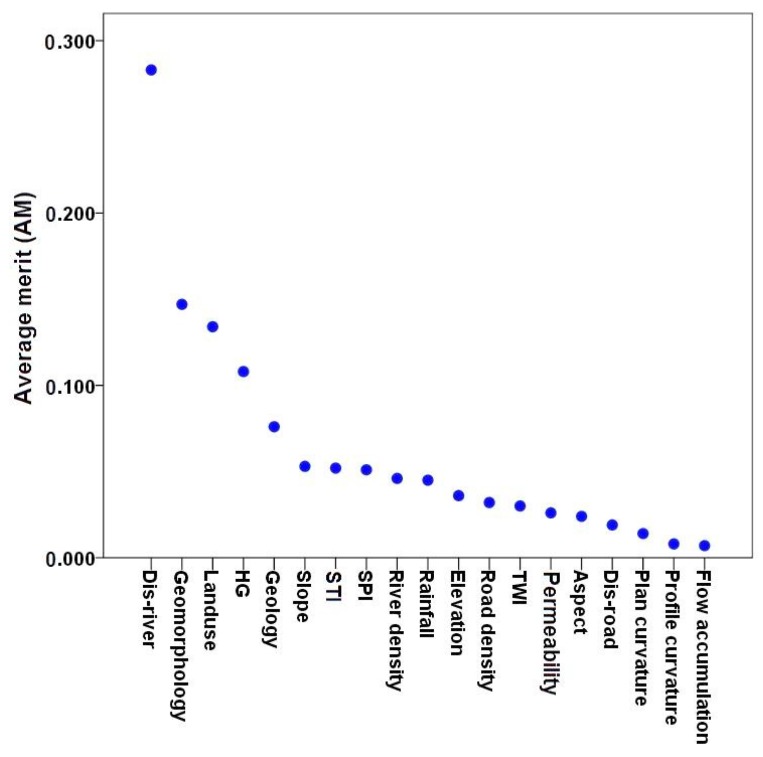
The most important conditioning factors for gully erosion modeling in the Klocheh Watershed, Kurdistan Province, Iran.

The predictive average merit of gully erosion affecting factors by the IGR method is shown in Figure 4. Factor selection results showed that 19 out of 22 conditioning factors were capable of modeling gully erosion prediction (AM > 0). Distance to river has the highest average merit for gully modeling (AM = 0.283). It is because most gullies in the study area were located beside the river networks. It is followed by geomorphology (AM = 0.147), land use (AM = 0.134), HG (AM = 0.134), lithology (AM = 0.076), slope (AM = 0.053), STI (AM = 0.052), SPI (AM = 0.051), river density (AM = 0.046), rainfall (AM = 0.045), elevation (AM = 0.036), road density (AM = 0.032), TWI (AM = 0.03), permeability (AM = 0.026), aspect (AM = 0.024), distance to road (AM = 0.019), profile curvature (AM = 0.008) and flow accumulation (AM = 0.007).

### 5.2. Gully Modeling Procedure or Optimization

In the modeling process, the determination of optimum parameters values in all algorithms is a critical issue for achieving an algorithm with the highest goodness-of-fit and performance. The optimum parameters of the investigated models are shown in Table 2. Basically, the new hybrid RF-ADTree and soft computing benchmark models (NBMU, SVM-Polynomial, SVM-RBF, and LR) were built using 19 conditioning factors and training dataset for the spatial prediction of gullies. In this study, the optimum number of seed (from 1 to 10) and iteration (from 10 to 20 iterations) was obtained with various numbers of iterations and seeds versus AUC and RMSE for the training and validation of datasets under a trial and error procedure.

**Figure 5:**
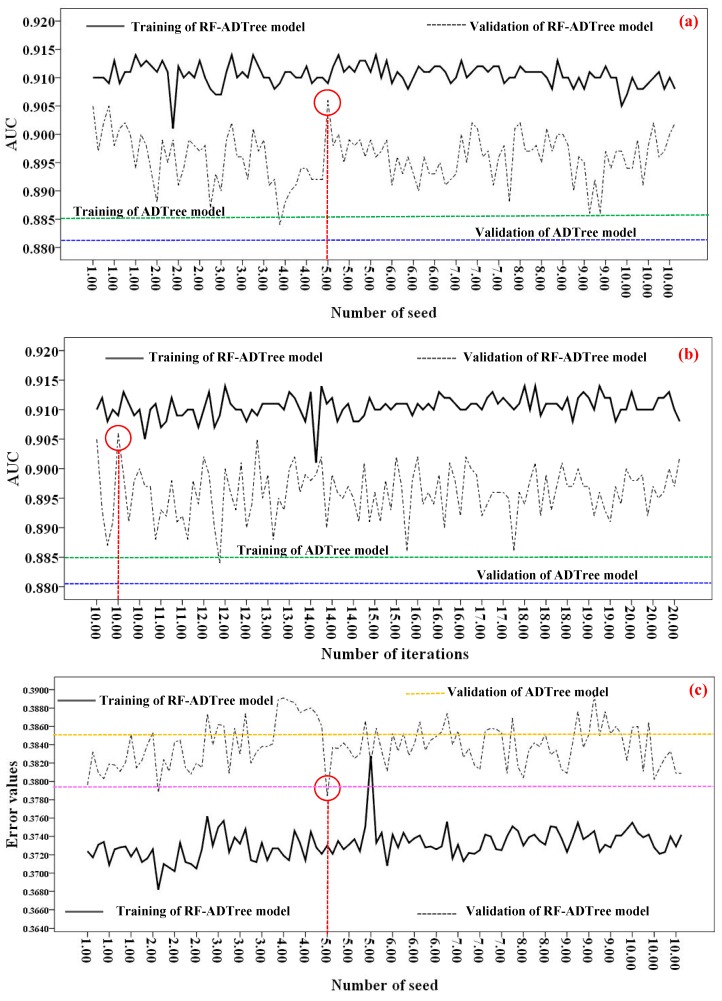

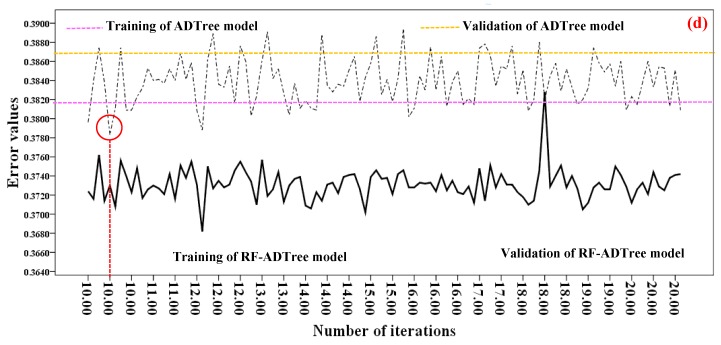
Modeling process for selecting the best values for the number of seed and iteration parameters for rotation forest (RF) as a Meta/ensemble classifier based on alternating decision tree (RF-ADTree) model: (**a**) number of seeds based on the area under the curve (AUC) of Receiver Operating Characteristic (ROC), (**b**) number of iterations based on the AUC of ROC, (**c**) number of seeds based on the root mean square error (RMSE), and (**d**) number of iterations based on the RMSE.

The results of optimum value selection for the number of seeds are shown in Figure 5a–d. The highest AUC values of RF-ADree model for the training and validation datasets (AUC = 0.906) were obtained with the number of seeds equal to 5 and the number of iterations equal to 10 (Figure 5a,b). Additionally, other results indicated that the lowest values of the RMSE (0.379) were obtained with the number of seeds and iterations equal to 5 and 10, respectively (Figure 5c,d).

The results of statistical performance analysis of models by the training dataset are shown in Table 3. These results indicate that all of the models have shown good performance for gully erosion in the training stage. In terms of sensitivity, the results stated that the new proposed model, RF-ADTree, has the highest sensitivity (0.877), indicating that 87.7% of gully erosions are correctly classified as gully erosion. It is followed by SVM-Polynomial kernel (0.875), SVM-RBF kernel (0.858), ADTree (0.842), LR (0.739) and the NBMU (0.768) models. Similarly, the values of specificity concluded that the new proposed model showed the highest value (0.804), followed by SVM-RBF kernel (0.793), ADTree (0.771), LR (0.764), SVM-Polynomial kernel (0.762) and the NBMU (0.747) models. The accuracy values indicated that the RF-ADTree model also has the highest value (0.837), followed by the SVM-RBF kernel (0.822), SVM-Polynomial kernel (0.809), ADTree (0.802), LR (0.797) and the NBMU (0.765) models. Additionally, the RF-ADTree hybrid model obtained the least RMSE value (0.373) in the training dataset, followed by the SVM-RBF kernel (0.375), LR (0.376), SVM-Polynomial kernel (0.378), ADTree (0.379) and the NBMU (0.398) models. Moreover, it can be observed that the RF-ADTree model has the highest AUC value (0.909), followed by the SVM-RBF kernel model (0.895), the ADTree (0.885), the LR model (0.876), the SVM-Polynomial kernel (0.871) and the NBMU model (0.844).

Performance analysis of the gully erosion models using validation dataset was also carried out (Table 4). The results showed that all models have shown high performance for prediction of gully erosion. Out of these, like the training stage, the RF-ADTree model has the highest predictive capability (sensitivity = 0.859; specificity = 0.795; accuracy = 0.824; RMSE = 0.378 and AUC = 0.926) and the NBMU model has shown the lowest performance (sensitivity = 0.756; specificity = 0.739; accuracy = 0.747; RMSE = 0.403 and AUC = 0.843). Other values of the statistical indices of model performance are shown in Table 4. Overall, the RF-ADTree model has the best performance for spatial prediction of gullies using both training and validation datasets. In other words, the RF model can improve the performance of ADTree as a base classifier for spatial prediction of gully erosion by detecting and eliminating the weakness of ADTree.

### 5.3. Development of Gully Erosion Maps

**Figure 6:**
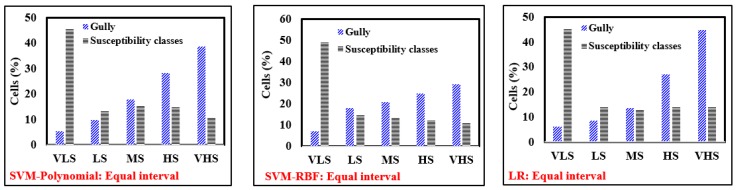

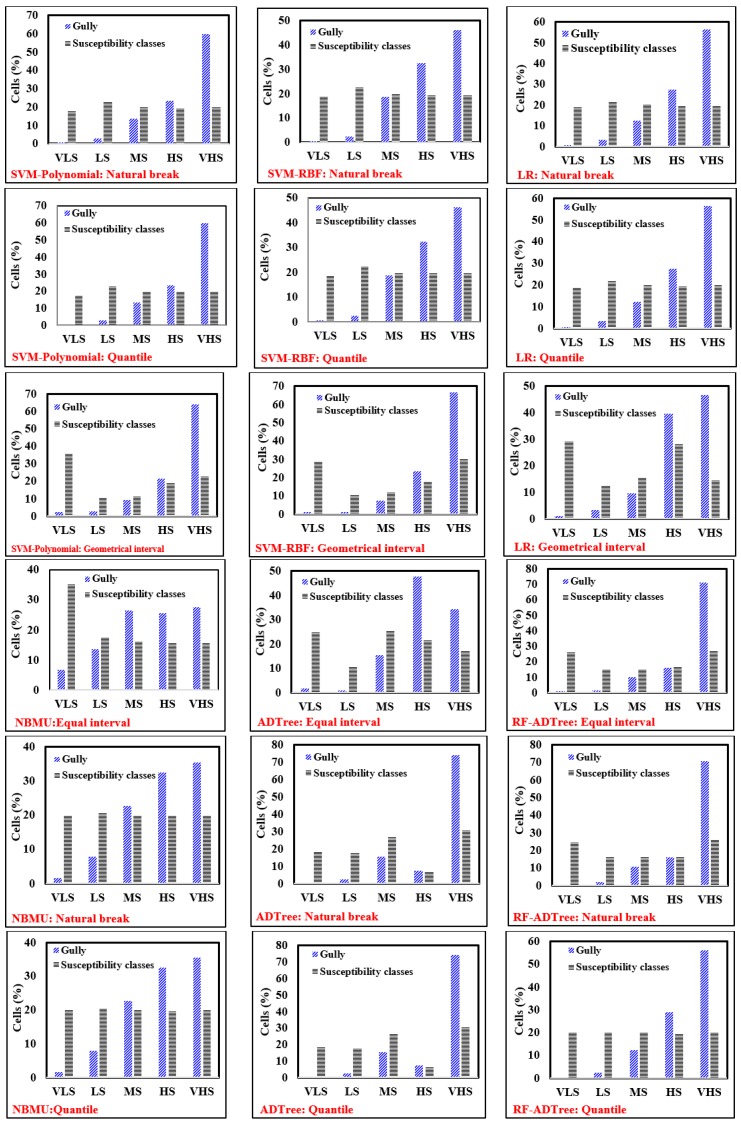

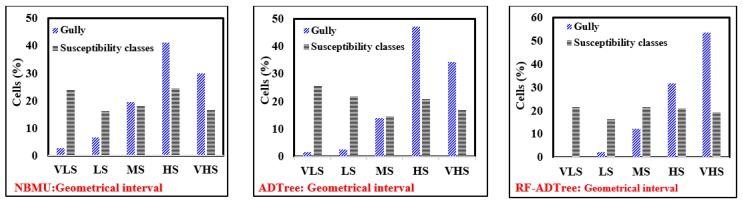
Histograms of all models for selecting the best classification method of gully susceptibility maps.

**Figure 7:**
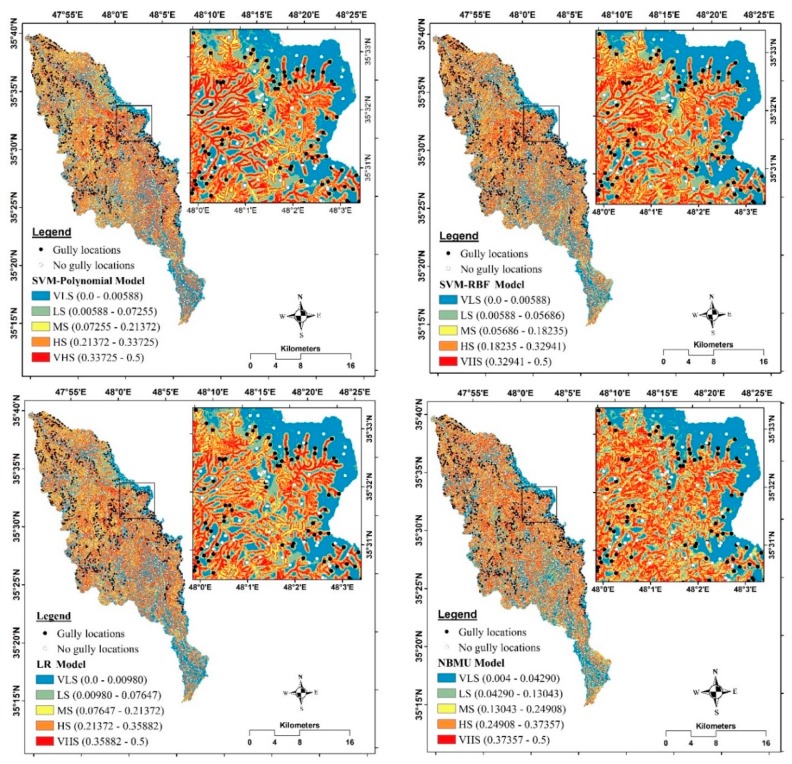

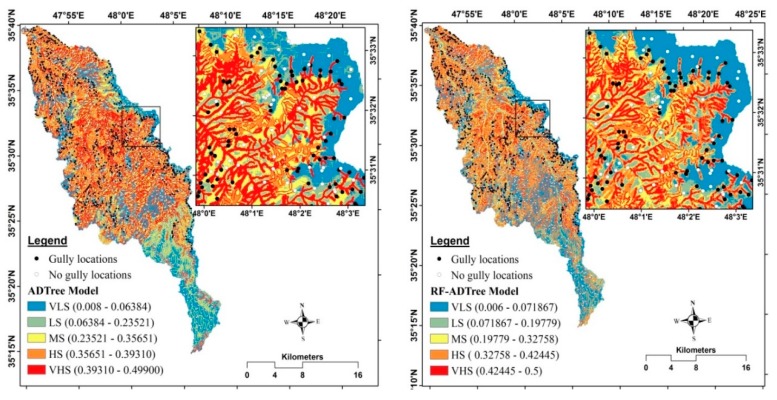
Gully erosion maps obtained by RF-ADTree model and other soft computing benchmark models.

As above-mentioned, the GESI for each cell converted into raster format and gully erosion susceptibility maps were prepared and they were classified. Generally, the histograms of all models for different classification methods indicated that the majority of the observed gullies are located in VHS class (Figure 6). According to the susceptibility map of the RF-ADTree model, the very high susceptibility class determined by equal interval, natural breaks, quantile and geometrical interval methods cover 26.9%, 26.4%, 20.2% and 19.5% of the whole watershed cells and, 71.4%, 70.6%, 56.1% and 53.4% of the observed gully cells, respectively. Therefore, for the RF-ADTree model, the equal interval method was selected as the most appropriate method for classification of gully erosion susceptibility. Accordingly, the geometrical interval method was selected for SVM-Polynomial kernel and SVM-RBF kernel susceptibility maps, and the natural break method was the appropriate classification method for the LR, the NBMU and the ADTree susceptibility maps. The gully erosion susceptibility maps generated by the developed models are shown in Figure 7.

### 5.4. The Contribution of the Sixth Most Important Factors Using GESMs

**Figure 8:**
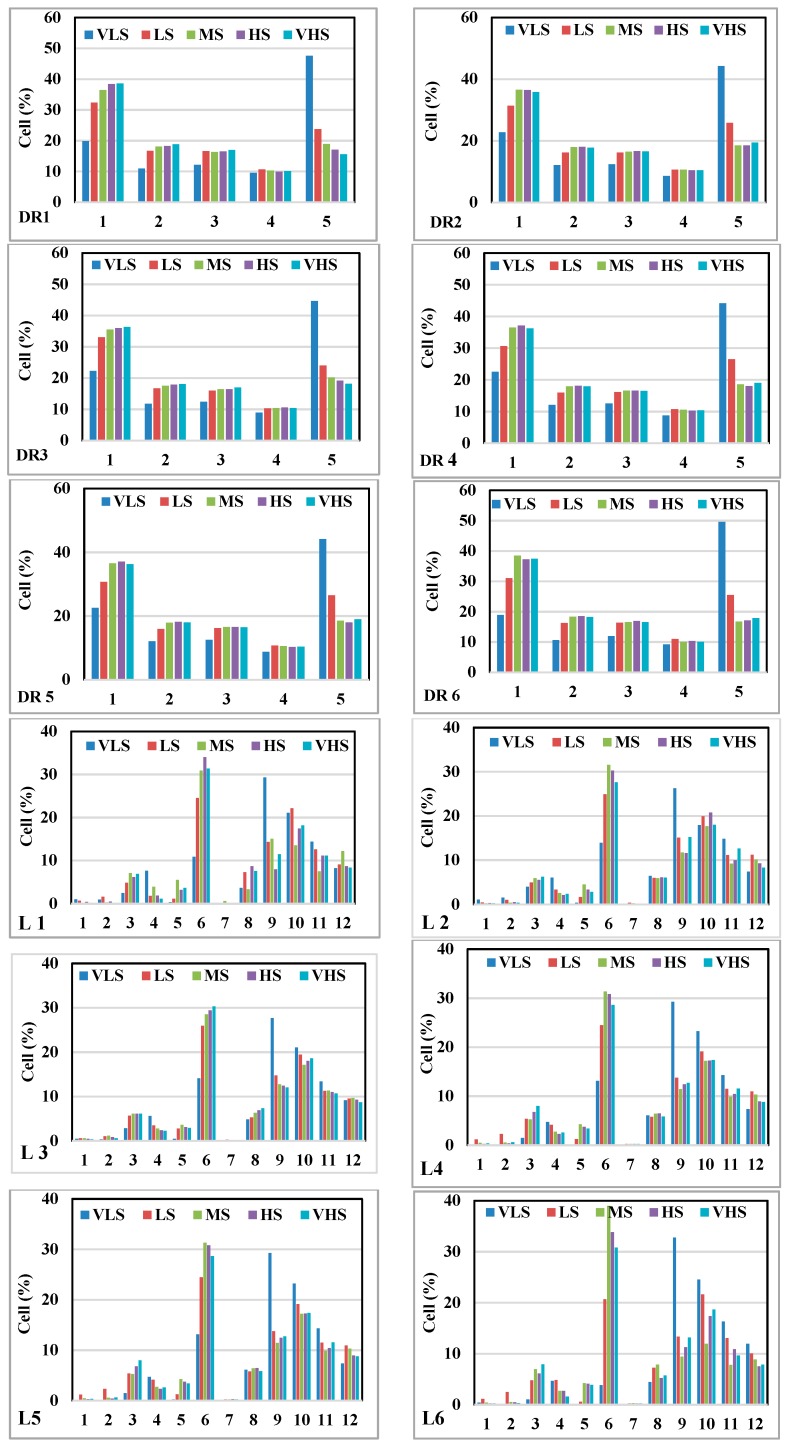

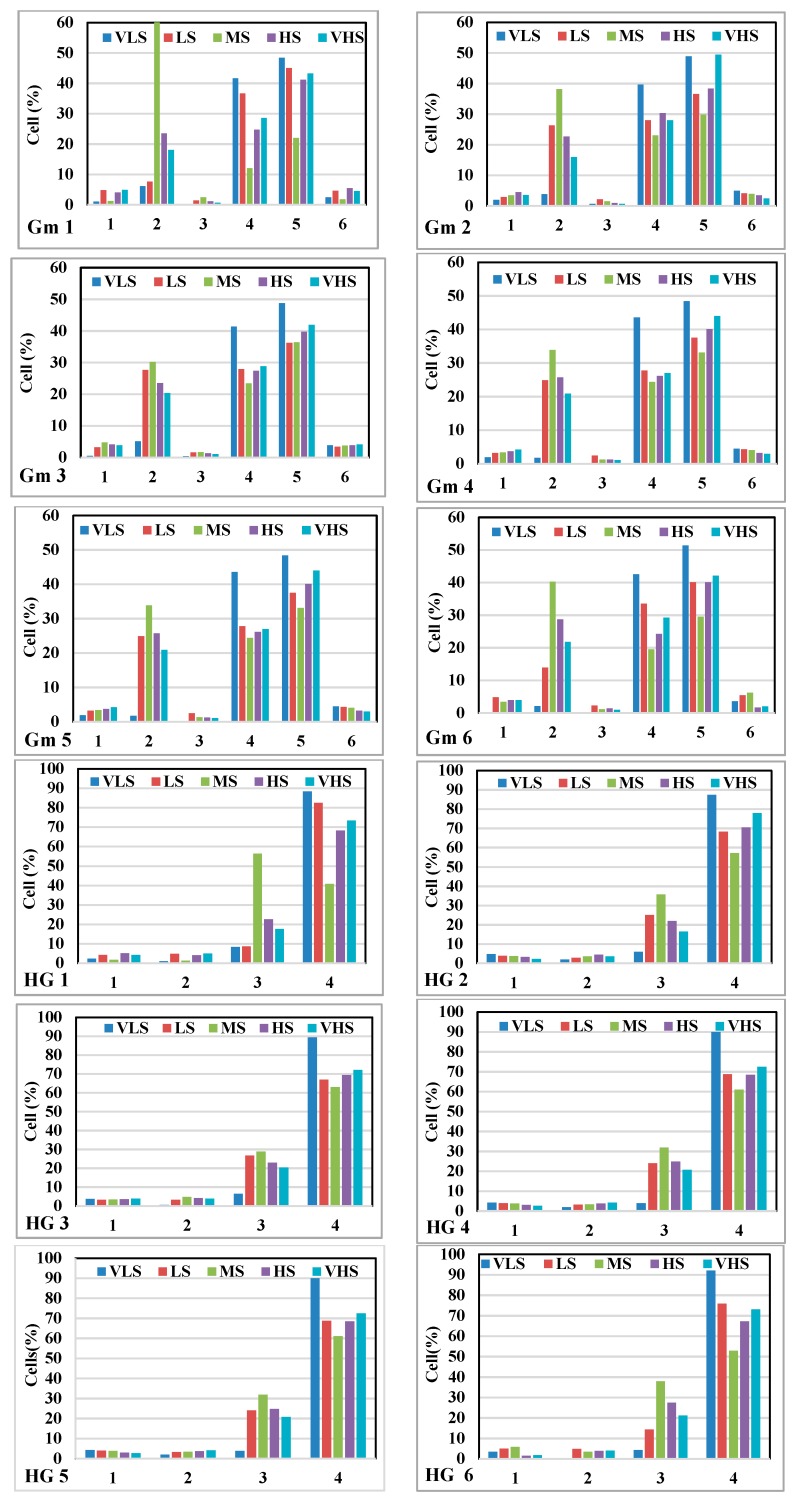

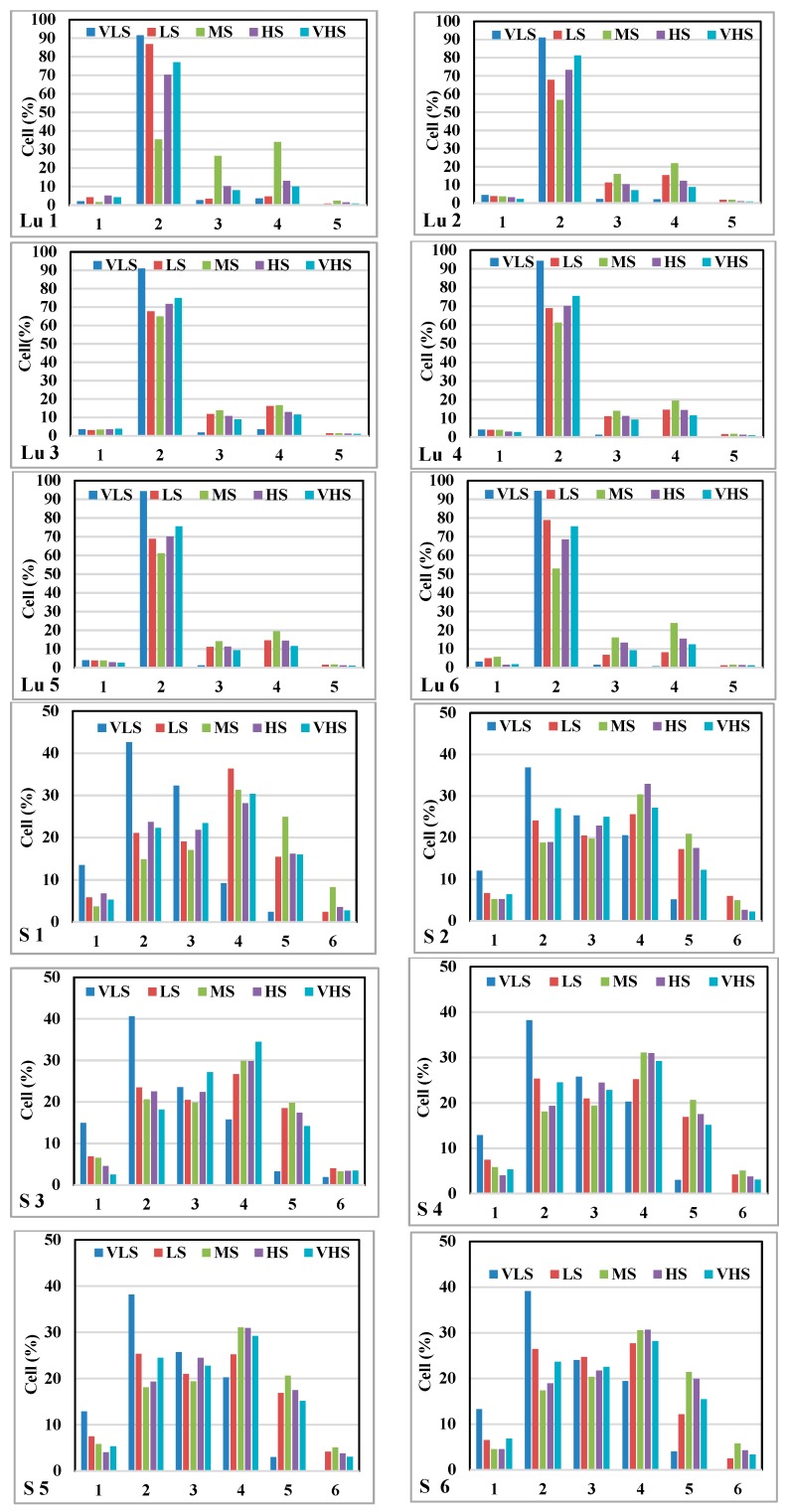
Histograms of gully susceptibility classes with the sixth most important factors: DR: distance to river; L: lithology; Gm: geomorphology; HG: hydrological group; Lu: land use; S: slope, 1: ADTree model; 2: Logistic Regression (LR) model; 3: Naïve Bayes Multinomial Updatable (NBMU) model; 4: Support Vector Machine-Radial Base Function (SVM-RBF) kernel model; 5: SVM-Polynomial kernel model and 6: RF-ADTree model.

In this study, we overlaid the sixth most important factors obtained by the IGR technique with gully erosion susceptibility maps developed by the models. The results are shown in Figure 8. It can be concluded that the first class of distance to river factor (<20 m) occupied the most cells of VHS class of gully erosion susceptibility map prepared by the ADTree (35.56%) model, followed by the RF-ADTree (37.39%), the NBMU (36.31%), the SVM-RBF kernel (36.26%), the SVM- Polynomial kernel (36.19%) and the LR (35.84%) models. It implied that the lowest distance from the rivers had the highest potential for gully erosion occurrence. Additionally, results indicated that the third class of geomorphology (fluvial sediment) occupied the most cells of VHS class in the LR (49.45%) model. It was followed by the SVM-RBF kernel (43.97%), the ADTree (43.25%) model, the RF-ADTree (42.11%), the NBMU (41.89%) and the SVM-Polynomial kernel (23.46%) models. It can be indicated that the fluvial sediments were more prone to gully occurrence in comparison to other geomorphologic classes. In terms of land use analysis, the results revealed that the dry-farming and cultivated lands covered the most cells of VHS class in the LR (81.21%) model while the lowest one was obtained for the NBMU (74.86%) model. Moreover, the ADTree, the RF-ADTree, the SVM-Poly kernel, and the SVM-RBF kernel models had the values of 77.05%, 75.52%, 78.77%, and 75.47%, respectively. The obtained results indicated that land use change in the study area was one of the principal reasons for gully erosion so that most of very high susceptibility class of gully susceptibility maps occurred on this land use unit. Among the four classes of soil hydrological groups (SHG), type D was more effective for gully erosion incidence in which results of overlaying the VHS class of susceptibility maps with SHG pinpointed that the most cells of VHS classes were obtained in the LR model (77.82%), followed by the SVM-Polynomial kernel (73.89%), the ADTree (73.24%), the RF-ADTree (73.06%), the SVM-RBF kernel (72.45%) and the NBMU (72.06%) models. In terms of lithology (Plm), results stated that the ADTree (31.35%) and the RF-ADTree (30.81%) models assigned the most cells of VHS class while the NBMU (30.29%), the SVM-RBF kernel (28.63%), the LR (27.59%) and the SVM-Polynomial kernel (26.72%) gained the other ranks. It implied also that the Plm lithological unit among other units was more responsible for gully erosion in the study area. In the case of slope angle (10–15°), results illustrated that the NBMU model (30.36%) had the highest value of the VHS class of gully susceptibility map. It was followed by the ADTree (30.36%), the SVM-RBF kernel (29.20%), the RF-ADTree (28.20%), the LR (27.12%) and the SVM-Polynomial kernel (26.26%) models. Overall, the findings indicated that the first class of distance to river, fluvial sediment, dry-farming and cultivated land, soil hydrological group D, Plm lithological unit and slope between 10° and 15° were more considerable for management and any prevention practice in the land allocation of the study area.

### 5.5. Evaluation and Comparison of Gully Erosion Maps

**Figure 9:**
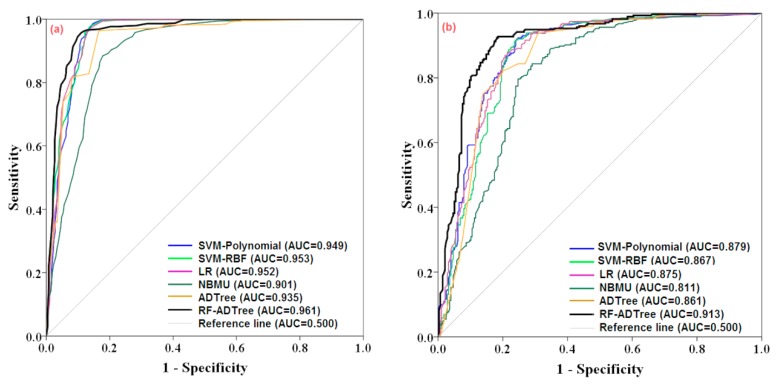
Model comparison using ROC (**a**) and AUC (**b**).

The new ensemble RF-ADTree model performance in prediction of gully erosion susceptibility was compared with SVM-Polynomial kernel, SVM-RBF kernel, LR, NBMU and ADTree benchmark models using ROC, gully density method, Friedman’s and Wilcoxon signed-rank test measures. The model accuracy was evaluated using the area under the ROC curve (AUC) for both training and validation datasets. In the training stage, the AUC of the ensemble RF-ADTree model had the highest value (AUC = 0.961), followed by the SVM-RBF kernel (AUC = 0.953), the LR (AUC = 0.952), the SVM-Polynomial kernel (AUC = 0.949), the ADTree (AUC = 0.935) and the NBMU model (AUC = 0.901) (Figure 9a).

Additionally, in the validation stage, the excellent predictive performance was taken place by the RF-ADTree that by the AUC equal to 0.913 indicating an accuracy of 91.3%. It is followed by the SVM-Polynomial kernel (AUC = 0.879), the LR (AUC = 0.875), the SVM-RBF kernel (AUC = 0.867), the ADTree (AUC = 0.861) and the NBMU model (AUC = 0.811) (Figure 9b). The above-mentioned results indicated that, similar to the RF-ADTree model, the other models had an acceptable accuracy in both training and validation stages.

The gully erosion density (GED) is another index in order to evaluate the reliability of gully erosion susceptibility maps. The gully erosion density increases for a perfect gully erosion susceptibility map from very low to very high susceptibility classes.. For the RF-ADTree model, the GED values were calculated equal to 0.003, 0.011, 0.046, 0.105 and 0.212 for VLS, LS, MS, HS and VHS gully erosion classes, respectively. Thus, it can be concluded that the RF-ADTree model generated an ideal gully erosion susceptibility map. For the SVM-Polynomial kernel model, the results indicated that the VHS class showed the highest GED (0.211), followed by the HS (0.082), MS (0.060), LS (0.021), and VLS (0.005) susceptibility classes. In the case of SVM-RBF kernel, the GED values were 0.003, 0.009, 0.048, 0.098 and 0.163 for VLS, LS, MS and VHS classes, respectively. Additionally, for the LR model, these values were obtained as 0.003, 0.011, 0.046, 0.105 and 0.212, respectively. In the case of the ADTree model, gully density for the VLS, LS, MS and VHS classes were calculated as 0.002, 0.011, 0.043, 0.082 and 0.180, respectively. In terms of the NBMU model, the values of 0.006, 0.029, 0.084, 0.123 and 0.133 were acquired for the VLS, LS, MS and VHS classes, respectively. Overall, all these models had an increasing trend in the value of GED from VLS to VHS classes.

### 5.6. Statistical Tests

The Friedman and Wilcoxon signed-rank tests were applied to evaluate the significant difference between the predictions of the gully erosion susceptibility models. Based on the Friedman’s test, in the study area, the average ranking was 4.80, 4.65, 3.49, 3.06, 2.71 and 2.29 for the RF-ADTree, ADTree, NBMU, LR, SVM-RBF kernel and SVM-Polynomial kernel models, respectively. Additionally, the chi-square statistic was 2040 at the 0.01 significance level, indicating a significant difference between the models (Table 5). Since the Friedman’s test is not capable of finding which model makes any difference when there is a significant difference, the Wilcoxon signed-rank test was used for pairwise comparing between the models. According to this test, between all gully erosion susceptibility models, the *p*-values had significant levels less than 5% and *z*-values were more than the critical values (–1.96 and +1.96) except between the ADTree and RF-ADTree models (*p*-value = 0.538 and *z*-value = −0.616). Among the pairwise comparisons with a significant difference, the LR and NBMU models had a significant difference at the level of 5%, the other pairs had significant difference at the level of 1% (Table 6). Accordingly, it can be concluded that the efficiency of all gully erosion susceptibility models had statistical differences with the others except the ADTree and RF-ADTree models which had a similar efficiency.

## 6. Discussion

Since gully erosion is considered as one of the main sources of sediments [6] and due to its different onsite and offsite effects [114], detection of areas that are more prone to gully erosion is an important strategy for preventing land degradation and soil transportation to rivers. In this study, main streams and their tributaries of the watershed were recognized and mapped using a new proposed and state-of-the-art ensemble algorithm namely the RF-ADTree model. Although, some conditioning factors can affect the development of gullies, selecting the most important ones to enhance the performance of the modeling process using feature selection is undeniable and essential [115]. Basically, among 22 conditioning factors in this study based on the IGR technique, 19 factors were known to be more effective so that distance to river (the most important role), geomorphology, land use, SHG, geology and slope angle were the first six significant factors. Indeed, the water shear stress in the areas where lithology is more susceptible to erosion with low permeability, mainly quaternary depositions, is the main factor for occurring and developing gullies in the study area. Wijdenes et al. [116] have declared that land use changes and lithology were responsible for developing gullies in Guadalentin catchment, southeast Spain. Moreover, Arabameri et al. [117] evaluated land use/land cover, lithology and distance to roads as the most important factors for gully occurrence in their study area. Rahmati et al. [22] based on the learning vector quantization (LVQ), pinpointed that distance to river, drainage density and land use are the most effective factors for the development of gullies. Most of gullies in the study area occurred along with the rivers and other factors were played as triggered factors such geomorphology and land use. Chaplot et al. [28] reported that the land use is a triggered factor for gully occurring.

The results of modeling process and gully susceptibility mapping evaluation using the new proposed model and some soft computing benchmark models such as NBMU, SVM-Polynomial kernel, SVM-RBF kernel, LR and ADTree indicated that the RF meta classifier combined with the ADTree algorithm, acquired the most goodness-of-fit and also performance using training and validation datasets. However, the ability of all these machine learning algorithms based on some statistical measures indicted that they were more successful for detection of the areas prone to gully erosion with emphasis on the new proposed model of RF-ADTree. Literature reviews showed that there is no study about the application of RF as a Meta classifier on gully erosion modeling; however, RF has been used more in landslide events as one of the soil erosion forms. Accordingly, results indicated that it had a high performance, for example, Pham et al. [118] revealed that the RF-Naïve Bayes (RFNB), Chen et al. [45] stated that the RF-Naïve Bayes Tree (RFNBT) and also Pham et al. [119] depicted that the RF based Functional Tree (RFFT) as a new and promising technique was more powerful technique and those outperformed the other Meta classifiers for landslide susceptibility modeling. Hong et al. [63] exploited some meta classifiers on the J48 Decision Tree (JDT) as a base classifier and concluded that the RFJDT model as a new proposed model had the highest performance in comparison to other models. Our findings can be explained that the RF Meta classifier uses feature extraction by principal component analysis (PCA) to optimize the learning of training dataset of the base classifier. This feature of the RF ensemble classifier leads to enhance the goodness-of-fit and also predictive ability of based classifier [64]. In other words, the RF model as a robust algorithm could be more efficient in reduction of both variance and bias of the base classier such as ADT in this study. The results also depicted that the ensemble models outperformed and outclassed the individual/single based classifiers. This is agreed and confirmed by Jebur et al. [120], Bui et al. [121], Bui et al. [107] and Shirzadi et al. [112]. Gully erosion susceptibility maps were prepared by all machine learning models used in this study and then classified using four known classification methods such as natural breaks, quantile, geometrical interval and equal interval [122]. All these methods for classifying gully erosion susceptibility maps and the results indicated that, for example natural breaks, geometrical interval and equal interval, had a low logical prediction as visualization. In other words, these classifications were led to an underestimate of prediction so that lower number of gully locations had occurred on high and very high susceptibility classes of gully erosion. Unlike, considering the histogram of gully distribution in this study revealed that the quantile method can be selected as the most appropriate method because of its higher conformity with the real ground condition than the other classifiers. Additionally, quantile method could assign more gully erosion location in the high and very high susceptibility classes of gully susceptibility maps in all machine learning algorithms. Some researchers have used the quantile classification method to divide the natural hazards susceptibility index such as Umar et al. [123], while Farncis et al. [122] used natural beaks, Pham et al. [124] used geometrical interval classification methods in their study.

The gully susceptibility maps were specified that the lowest distance from the rivers caused the most susceptibility to gully erosion. The results of the new proposed model of RF-ADTree was overlaid with the first six conditioning factors concluded that in terms of distance to river, the high (37.21%) and the very high (37.39%) susceptibility classes covered the most cells of gully erosion so that they were located on the first class of distance to river (<20 m). In terms of geomorphology, the fluvial sediment unit (quaternary deposition) mostly covered the high (40.11) and the very high (42.11) susceptibility classes of gully erosion susceptibility map. In terms of land use, according to the RF-ADTree model, dry-farming and cultivated lands were more susceptible to gully occurrence in which the high and the very high susceptibility classes occupied 68.45%, and 75.52%, respectively. Additionally, the high (67.22) and the very high (73.06) susceptibility classes of gully erosion map are corresponded with soil hydrologic group (SHG) D unit. It is noticed that soil hydrologic group D is mainly the soils that have very low permeability and infiltration rate when thoroughly wet resulting in a high runoff potential [125]. Therefore, soil hydrologic group D provided conditions for higher gully occurrence and development over the study area. Geologic analysis indicted that among 12 lithological units, the Plm unit had the highest susceptibility to gully erosion in which the most percentages of high (33.83%) and very high (30.81%) susceptibility classes were located in this lithological unit. The Plm as a low permeability unit consists of Pliocene marls including clay limestone, marl, sandstone, silty tuff, conglomerate, sandstone and travertine. This unit has mainly been covered by hilly slopes in the study area with an average elevation of 1800–2000 m. It generally has low slope and its color is often white to worm, pepper and sometimes red, dark gray and yellow. Slope angles were other important factors which slope angles between 2° and 15° were more significant for gully occurrence. However, slope angle between 15° and 20° covered the high (3.65) and the very high susceptibility classes in the study area. This class of slope angle dealt mainly with soil hydrologic groups C and D, Plm lithological unit, hilly mountain and fluvial sediment of geomorphology class under the dry farming and also cultivated land areas of land use factor. The validity of gully erosion susceptibility map prepared by the new proposed model in addition to the AUC, also was statistically checked and the results were verified and confirmed the applicability of this model and its prepared susceptibility map for gully management purposes.

## 7. Conclusions

Gully erosion as one of the soil threatening hazards leads to damage and destruction of infra-structure such as check dams in the Klocheh Watershed, Kurdistan Province, Iran, that was shown Figure 1. However, identification, prediction, prevention and management of gullies have always been top priorities for soil scientists, natural resources authorities and land managers. Therefore, an accurate spatial prediction of the gully erosion locations is an essential issue for conservation of natural resources such as soil and reducing its potential risks. For this purpose, we developed a new designed intelligence-based ensemble model named RF-ADTree which could successfully map the spatial prediction of gully erosion development in the Klocheh Watershed, Kurdistan Province, Iran. Additionally, we used of five soft computing benchmark models to check the goodness-of-fit and prediction accuracy of the new proposed model. Results of validation indicted that although all machine learning algorithms had high prediction accuracy; however, the new ensemble model was successful in gully erosion prediction due to the generation of a very accurate gully susceptibility map of the study area compared to other benchmark models. We recommend this model for gully modeling in other similar areas with caution since other conditioning factors might be responsible for gully erosion in other areas; while, distance to river was the most susceptible conditioning factor in the Klocheh watershed. Therefore, the obtained gully erosion map from the new developed model can be useful for planners, decision makers and engineers to better sustainably manage and decrease the damage and losses from the existing and future gullies, or also better manage the high and very high susceptible zones by appropriate decisions by preventive measures and mitigation procedures.

## Figures and Tables

**Table 1:** Gully conditioning factors and their classes for gully modeling in Klocheh Watershed, Kurdistan Province, Iran.

	No.	Factors	Classes	Classification Method
**Topographic**	1	Slope (o)	(1) 0–2; (2) 2–5; (3) 5–10; (4) 10–15; (5) 15–20; (6) >20	Manual
	2	Aspect	(1) Flat; (2) North; (3) Northeast; (4) East; (5) Southeast; (6) South; (7) Southwest; (8) West; (9) Northwest	Azimuth
	3	Elevation (m)	(1) 1612–1700; (2) 1700–1800; (3) 1800–1900; (4) 1900–2000; (5) 2000–2100; (6) 2100–2200; (7) 2200–2300; (8) 2300–2400	Manual
	4	Plan curvature (m^−1^)	(1) [(−5.67)–(−0.736)]; (2) [(−0.736)–(−0.188)]; (3) [(−0.188)–0.149]; (4) [0.149–0.697]; (5) [0.6974–5.08]	Natural break
	5	Profile curvature (m^−1^)	(1) [(−6.357)–(−0.972)]; (2) [(−0.972)–(−0.187)]; (3) [(−0.187)–0.317]; (4) [0.317–1.1]; (5) [1.1–7.94]	Natural break
	6	STI	(1) 0–1.286; (2) 1.286–2.894; (3) 2.894–5.145; (4) 5.145–8.468; (5) 8.468–27.33	Natural break
	7	VD	(1) 0–48.231; (2) 48.231–108.520; (3) 108.520–176.340; (4) 176.340–254.720; (5) 254.720–384.340	Natural break
**Hydrological**	8	Rainfall (mm)	(1) 261–286; (2) 286–298; (3) 298–306; (4) 306–312; (5) 312–322	Natural break
	9	SPI	(1) 0–112.4; (2) 112.4–224.8; (3) 224.8–401.5; (4) 401.5–722.7; (5) 722.7–4095	Natural break
	10	TWI	(1) 1–3; (2) 3–4; (3) 4–5; (4) 5–6; (5) 6–9.059	Natural break
	11	HG	(1) A; (2) B; (3) C; (4) D	HG type
	12	Flow accumulation	(1) 0–5; (2) 5–10; (3) 10–20; (4) 20–30; (5) >30	Manual
	13	Permeability	(1) Low; (2) Moderate; (3) High	Permeability type
	14	Distance to river (m)	(1) 0–20; (2) 20–40; (3) 40–60; (4) 60–80; (5) >80	Manual
	15	River density (km/km^2^)	(1) 0–2.775; (2) 2.775–4.810; (3) 4.810–6.598; (4) 6.598–8.694; (5) 8.694–15.72	Natural break
**Lithological**	16	Lithology	(1) JL; (2) JS; (3) M^m^; (4) PL^b^; (5) P^cg^; (6) Pl^m^; (7) Pl^t^; (8) Qa^l^; (9) Q^c^; (10) Qt^r^; (11) Qt^1^; (12) Qt^2^	Lithology type
	17	Distance to fault (m)	(1) 0–100; (2) 100–200; (3) 200–500; (4) 500–1000; (5) >1000	Manual
	18	Fault density (km/km^2^)	(1) 0–0.287; (2) 0.287–0.823; (3) 0.823–1.270; (4) 1.270–1.820; (5) 1.820–2.440	Natural break
**Land cover**	19	Land use	(1) Wood land; (2) Dry-farming and cultivated lands; (3) Poor pastures; (4) Semi-dense pastures; (5) Destroyed pastures	Land use type
**Anthropogenic**	20	Distance to road (m)	(1) 0–100; (2) 100–200; (3) 200–300; (4) 300–500; (5) >500	Manual
	21	Road density (km/km^2^)	(1) 0–0.684; (2) 0.684–1.750; (3) 1.750–2.570; (4) 2.570–3.690; (5) 3.690–6.980	Natural break
**Geomorphology**	22	Geomorphology	(1) The valley plain unit (2) Hilly unit; (3) Mountain unit; (4) New plain unit; (5) Old plain unit; (6) Fluvial sediment unit	Geomorphology type

**Table 2:** Machine learning algorithm used parameters for gully modeling in the Klocheh Watershed, Kurdistan Province, Iran.

Model Name	Description of Parameters
**RF-ADTree**	Classifier: ADTree; MaxGroup: 3; MinGroup: 3; Number of iterations: 10; Number of Groups: False; Projection Filter: PCA; Removed Percentage: 50; Number of seeds: 5
**ADTree**	Number of Boosting Iterations: 10; Random Seed: 0; Save Instance Data: false; Search Path: Expand all Paths
**LR**	Maximum Its: −1; Ridge: 1.0 × 10^8^
**SVM-PolyKernel**	Build Logistic Models: True; C: 1; Check turned Off: False; Epsilon: 1.0 × 10^12^: Filter Type: Not normalization/standardization; Kernel: PolyKernel; Number of folds: −1; Tolerance Parameter: 0.001
**SVM-RBF**	Build Logistic Models: True; C: 1; Check turned Off: False; Epsilon: 1.0 × 10^12^: Filter Type: Not normalization/standardization; Kernel: RBF; Number of folds: −1; Tolerance Parameter: 0.001
**NBMU**	-

**Table 3:** Model performances in the training dataset for the new hybrid “RF-ADTree” model and other benchmark models.

Measures	NBMU	SVM-Polynomial	SVM-RBF	LR	ADTree	RF-ADTree
True positive	466	461	494	470	476	501
True negative	513	574	558	550	551	570
False positive	174	179	146	170	164	139
False negative	127	66	82	90	89	70
Sensitivity (%)	0.786	0.875	0.858	0.839	0.842	0.877
Specificity (%)	0.747	0.762	0.793	0.764	0.771	0.804
Accuracy (%)	0.765	0.809	0.822	0.797	0.802	0.837
RMSE	0.398	0.378	0.375	0.376	0.379	0.373
AUC	0.844	0.871	0.895	0.876	0.885	0.909

**Table 4:** Model performances in the validation dataset for the new hybrid “RF-ADTree” model and other benchmark models.

Measures	NBMU	SVM-Polynomial	SVM-RBF	LR	ADTree	RF-ADTree
True positive	201	195	198	201	204	213
True negative	210	244	227	236	240	240
False positive	74	80	77	47	71	62
False negative	65	31	48	39	35	35
Sensitivity (%)	0.756	0.863	0.805	0.838	0.854	0.859
Specificity (%)	0.739	0.753	0.747	0.834	0.772	0.795
Accuracy (%)	0.747	0.798	0.773	0.836	0.807	0.824
RMSE	0.403	0.380	0.381	0.380	0.384	0.378
AUC	0.843	0.863	0.873	0.869	0.882	0.906

**Table 5:** Average ranking of the five gully erosion models for the study area using the Friedman’s test.

No.	Gully Models	Mean Ranks	χ2	Sig.
1	SVM-Polynomial	2.29	2040	0.000
2	SVM-RBF	2.71		
3	LR	3.06		
4	NBMU	3.49		
5	ADTree	4.65		
6	RF-ADTree	4.80		

**Table 6:** Performance of the RF-ADTee model compared to other gully erosion models using Wilcoxon signed-rank test (two-tailed).

No.	Pairwise Comparison	NPD	NND	*z*-value	*p*-value	Significance
**1**	SVM-Polynomial vs. SVM-RBF	303	540	−9.755	0.000	Yes
**2**	SVM-Polynomial vs. LR	245	700	−13.424	0.000	Yes
**3**	SVM-Polynomial vs. NBMU	349	905	−9.343	0.000	Yes
**4**	SVM-Polynomial vs. ADTree	196	1057	−23.838	0.000	Yes
**5**	SVM-Polynomial vs. RF-ADTree	129	1126	−26.125	0.000	Yes
**6**	SVM-RBF vs. LR	325	568	−4.621	0.000	Yes
**7**	SVM-RBF vs. NBMU	434	813	−3.536	0.000	Yes
**8**	SVM-RBF vs. ADTree	234	1009	−21.050	0.000	Yes
**9**	SVM-RBF vs. RF-ADTree	194	1049	−23.189	0.000	Yes
**10**	LR vs. NBMU	448	780	−2.020	0.043	Yes
**11**	LR vs. ADTree	273	978	−19.344	0.000	Yes
**12**	LR vs. RF-ADTree	222	1019	−21.772	0.000	Yes
**13**	NBMU vs. ADTree	291	916	−19.038	0.000	Yes
**14**	NBMU vs. RF-ADTree	249	919	−19.714	0.000	Yes
**15**	ADTree vs. RF-ADTree	578	591	−0.616	0.538	No

NPD: Number of positive; NND: Number of negative differences.
